# Phase analysis of circadian-related genes in two tissues

**DOI:** 10.1186/1471-2105-7-87

**Published:** 2006-02-23

**Authors:** Delong Liu, Shyamal D Peddada, Leping Li, Clarice R Weinberg

**Affiliations:** 1 Biostatistics Branch, National Institute of Environmental Health Sciences, MD: A3-03, 111 TW Alexander Dr, Research Triangle Park, NC 27709, USA; 2 CIIT Centers for Health Research, 6 Davis Drive, Research Triangle Park, NC, 27709, USA

## Abstract

**Background:**

Recent circadian clock studies using gene expression microarray in two different tissues of mouse have revealed not all circadian-related genes are synchronized in phase or peak expression times across tissues *in vivo*. Instead, some circadian-related genes may be delayed by 4–8 hrs in peak expression in one tissue relative to the other. These interesting biological observations prompt a statistical question regarding how to distinguish the synchronized genes from genes that are systematically lagged in phase/peak expression time across two tissues.

**Results:**

We propose a set of techniques from circular statistics to analyze phase angles of circadian-related genes in two tissues. We first estimate the phases of a cycling gene separately in each tissue, which are then used to estimate the paired angular difference of the phase angles of the gene in the two tissues. These differences are modeled as a mixture of two von Mises distributions which enables us to cluster genes into two groups; one group having synchronized transcripts with the same phase in the two tissues, the other containing transcripts with a discrepancy in phase between the two tissues. For each cluster of genes we assess the association of phases across the tissue types using circular-circular regression. We also develop a bootstrap methodology based on a circular-circular regression model to evaluate the improvement in fit provided by allowing two components versus a one-component von-Mises model.

**Conclusion:**

We applied our proposed methodologies to the circadian-related genes common to heart and liver tissues in Storch *et al*. [2], and found that an estimated 80% of circadian-related transcripts common to heart and liver tissues were synchronized in phase, and the other 20% of transcripts were lagged about 8 hours in liver relative to heart. The bootstrap *p*-value for being one cluster is 0.063, which suggests the possibility of two clusters. Our methodologies can be extended to analyze peak expression times of circadian-related genes across more than two tissues, for example, kidney, heart, liver, and the suprachiasmatic nuclei (SCN) of the hypothalamus.

## Background

Circadian rhythms (or the biologic clocks that control them) have stimulated interest in recent years due to their importance in orchestrating physiological behavior, biological processes, and adaptability of biological systems to changes in environment [1-3]. Many circadian-related genes have been explored using high-throughput DNA microarray technology [1-3]. These studies also have stimulated efforts to apply and develop methodologies in circular/directional statistics to elucidate important characteristics of circadian gene expression and also compare their patterns of peak expression times (phase angles) across different tissue types, to help elucidate their diverse tissue-specific functions [1,2,4,5].

As periodic oscillation characterizes the expression pattern of both circadian genes and cell cycle genes, many correlation-based and Fourier-based methodologies [1-3] proposed for analyzing cell cycle gene expression can be directly applied to circadian gene expression analysis. However, there are some distinct differences between studies in cell cycle gene expression and circadian gene expression. First, most cell cycle gene expression patterns are based on cell cultures studied in *vitro*, while most circadian gene expressions are based on various tissues or organs in *vivo*. Consequently, circadian gene expression may be more complex or tissue/cell-specific. Second, the four phases of a cell cycle, namely, *G*_1_, *S*, *G*_2_, and *M *phases, have been well characterized through intensive research over the last thirty years, and more than 54 mammalian [6] and 104 yeast cell cycle genes have been identified [7]. In contrast, to date, less is known about circadian genes: only eight core mammalian circadian genes have been identified: *Csnk1e*, *Cry1*, *Cry2*, *Per1*, *Per2*, *Per3*, *Clock*, and *Bmall *[8]. In addition, it is not clear whether these known circadian genes and any other circadian-related genes identified from high-throughput microarrays can be assigned to a few functional phases, in analogy to the phases (*G*_1_, *S*, *G*_2_, *M*) in cell cycle. Note that many studies on cell cycle gene expression based on microarray were on same organism [6] and cell-type [7] under different experimental conditions. Therefore, we expect that a set of cell cycle genes commonly expressed in various conditions are consistent in their peak expression/activation time [9]. However, it is an opening question whether phases or peak expression times for a set of circadian-related genes commonly expressed in multiple tissues, such as heart, liver, kidney, and SCN of the hypothalamus are in synchrony because expression of some circadian-related genes may be tissue-specific. Statistical tools for analyzing such a type of circular data cross multiple tissues need to be developed.

The phase angles estimated from cycling transcripts in Panda *et al*. [1] and Storch *et al*. [2] can be regarded as points on a circle of unit radius, which are treated as circular data in circular/directional statistics [10,11]. Circular data are commonly modeled with a von Mises distribution function on the unit circle, an analog to the normal distribution for linear data. The main feature of circular data is that it is directional and classical methods based on linear data can produce meaningless results. As an example, suppose a bird takes off in the northeast direction at an angle of 2°, while another takes off in the southeast direction at an angle 358° then their mean direction (by usual linear methods) is 180°, or due west! Means and variances and other statistical analyses must respect the directional nature of the data to avoid such nonsensical results. For example, a sum of two points on a unit circle is calculated as the sum of the two vectors, yielding a vector with certain direction and length. With this vector averaging, the two birds now have a mean direction of 0°, corresponding properly to due east. Special methods are available in the literature for describing correlation and regression between circular variables [10,11]. One needs to be cautious when analyzing circular and linear variables simultaneously. In some cases circadian phase angles have been mistreated as linear variables in linear regression of the phase angle on the period and amplitude [12]. Results so obtained may not be useful for interpretation.

The motivation for our work is based on the observation in [1-3] that some circadian-related genes that are expressed in two tissues are systematically lagged in peak expression time in the two tissues. Panda *et al*. [1] reported that many of the 28 circadian-related transcripts common to the suprachiasmatic nuclei (SCN) of the hypothalamus and liver, including *Per2 *and *Rev-Erbβ*, are delayed by 4–8 hrs in peak expression in liver relative to the SCN. Ueda *et al*. [3] validated *in vitro *that the *Rev-ErbA/ROR *response element in both the SCN and liver tissues is expressed in phase with *Bmal1 *and in anti-phase with *Per2 *oscillation. These studies suggest that the coordinated temporal expression of circadian genes in-phase and anti-phase in different tissues is an interesting but a complex biological phenomenon. Statistical analysis tools for studying this type of interesting biological questions arising in recent genomics studies are needed.

To address the above questions, we propose a few steps in the following sections. Given a set of circadian-related genes common to two tissues, we first fit a random-periods model [13] to the time-course expression for each gene individually in each of two tissues, to estimate its phase angles along with periods and amplitudes. The angular difference between the two phases for each gene can be represented as an angle, i.e. as a point on a circle. Using a mixture of two von Mises distributions, we cluster angular differences of the genes into two groups; genes whose expressions are synchronized (mean difference is close 0) in the two tissues and those whose expressions are different in the two tissues. The identified clusters may provide a hint on association of circadian genes specific to these tissues. We then assess the association of each set of genes common to two tissues using Down and Mardia's circular-circular regression model [14]. In addition, we propose a new circular-circular regression-based bootstrap method to assess the mixture of two homogeneous phase distributions for the two tissues. We illustrate the proposed methodologies using the heart and liver circadian-related gene expression data sets from Storch *et al*. [2].

## Methods

### Phase estimation

One characterizing feature of circadian-related genes expression is the periodically oscillating pattern. Sinusoidal functions have been used to model the circadian gene expression level [1-3]. We apply the "random-periods model" [13] to estimate the phase angles, period, and amplitude together for a given circadian gene expression using nonlinear least-squares regression. While there is no attenuation in circadian gene expression, the sinusoidal component of the "random-periods model" is reduced to a simple sinusoidal function *K*_*g*_cos(2*πt*/*T*_*g *_+ *φ*_*g*_), where *K*_*g*_, *T*_*g*,_, and *φ*_*g *_are the amplitude, period, and phase (angle) of gene *g*. The phase parameter *φ*_*g *_indicates when the expression of the *g *gene reaches its maximum.

### A mixture of two von Mises distributions for circular paired-difference data

After estimation of activation times or phase angles for a set of circadian-related genes that expressed in two tissues or organs, we are interested in examining whether the phase angles are synchronous or not. Let φ^gx
 MathType@MTEF@5@5@+=feaafiart1ev1aaatCvAUfKttLearuWrP9MDH5MBPbIqV92AaeXatLxBI9gBaebbnrfifHhDYfgasaacH8akY=wiFfYdH8Gipec8Eeeu0xXdbba9frFj0=OqFfea0dXdd9vqai=hGuQ8kuc9pgc9s8qqaq=dirpe0xb9q8qiLsFr0=vr0=vr0dc8meaabaqaciaacaGaaeqabaqabeGadaaakeaaiiGacuWFgpGzgaqcamaaDaaaleaacqWGNbWzaeaacqWG4baEaaaaaa@3179@ and φ^gy
 MathType@MTEF@5@5@+=feaafiart1ev1aaatCvAUfKttLearuWrP9MDH5MBPbIqV92AaeXatLxBI9gBaebbnrfifHhDYfgasaacH8akY=wiFfYdH8Gipec8Eeeu0xXdbba9frFj0=OqFfea0dXdd9vqai=hGuQ8kuc9pgc9s8qqaq=dirpe0xb9q8qiLsFr0=vr0=vr0dc8meaabaqaciaacaGaaeqabaqabeGadaaakeaaiiGacuWFgpGzgaqcamaaDaaaleaacqWGNbWzaeaacqWG5bqEaaaaaa@317B@ denote the estimated phase angles of a circadian-related gene *g, g *= *1, 2, ..., n*, in the two tissues *x *and *y*, where -*π*≤ φ^gx
 MathType@MTEF@5@5@+=feaafiart1ev1aaatCvAUfKttLearuWrP9MDH5MBPbIqV92AaeXatLxBI9gBaebbnrfifHhDYfgasaacH8akY=wiFfYdH8Gipec8Eeeu0xXdbba9frFj0=OqFfea0dXdd9vqai=hGuQ8kuc9pgc9s8qqaq=dirpe0xb9q8qiLsFr0=vr0=vr0dc8meaabaqaciaacaGaaeqabaqabeGadaaakeaaiiGacuWFgpGzgaqcamaaDaaaleaacqWGNbWzaeaacqWG4baEaaaaaa@3179@ ≤*π*, -*π *≤φ^gy
 MathType@MTEF@5@5@+=feaafiart1ev1aaatCvAUfKttLearuWrP9MDH5MBPbIqV92AaeXatLxBI9gBaebbnrfifHhDYfgasaacH8akY=wiFfYdH8Gipec8Eeeu0xXdbba9frFj0=OqFfea0dXdd9vqai=hGuQ8kuc9pgc9s8qqaq=dirpe0xb9q8qiLsFr0=vr0=vr0dc8meaabaqaciaacaGaaeqabaqabeGadaaakeaaiiGacuWFgpGzgaqcamaaDaaaleaacqWGNbWzaeaacqWG5bqEaaaaaa@317B@ ≤ *π*. Further, we model the distribution of the angular difference

Δg=φ^gy−φ^gx, −π≤Δg≤π     (1)
 MathType@MTEF@5@5@+=feaafiart1ev1aaatCvAUfKttLearuWrP9MDH5MBPbIqV92AaeXatLxBI9gBaebbnrfifHhDYfgasaacH8akY=wiFfYdH8Gipec8Eeeu0xXdbba9frFj0=OqFfea0dXdd9vqai=hGuQ8kuc9pgc9s8qqaq=dirpe0xb9q8qiLsFr0=vr0=vr0dc8meaabaqaciaacaGaaeqabaqabeGadaaakeaacqqHuoardaWgaaWcbaGaem4zaCgabeaakiabg2da9GGaciqb=z8aMzaajaWaa0baaSqaaiabdEgaNbqaaiabdMha5baakiabgkHiTiqb=z8aMzaajaWaa0baaSqaaiabdEgaNbqaaiabdIha4baakiabcYcaSiabbccaGiabgkHiTiab=b8aWjabgsMiJkabfs5aenaaBaaaleaacqWGNbWzaeqaaOGaeyizImQae8hWdaNaaCzcaiaaxMaadaqadaqaaiabigdaXaGaayjkaiaawMcaaaaa@4B54@

as a mixture of two von Mises distributions. One component will correspond to a subset of the *n *genes have the same phase angle in the two tissues and the other will correspond to genes having unequal phase angles in the two tissues. Thus the probability density function is given by:

f(Δg)=∑i=12pifi(Δg), ∑i=12pi=1,     (2)
 MathType@MTEF@5@5@+=feaafiart1ev1aaatCvAUfKttLearuWrP9MDH5MBPbIqV92AaeXatLxBI9gBaebbnrfifHhDYfgasaacH8akY=wiFfYdH8Gipec8Eeeu0xXdbba9frFj0=OqFfea0dXdd9vqai=hGuQ8kuc9pgc9s8qqaq=dirpe0xb9q8qiLsFr0=vr0=vr0dc8meaabaqaciaacaGaaeqabaqabeGadaaakeaacqWGMbGzcqGGOaakcqqHuoardaWgaaWcbaGaem4zaCgabeaakiabcMcaPiabg2da9maaqahabaGaemiCaa3aaSbaaSqaaiabdMgaPbqabaGccqWGMbGzdaWgaaWcbaGaemyAaKgabeaaaeaacqWGPbqAcqGH9aqpcqaIXaqmaeaacqaIYaGma0GaeyyeIuoakiabcIcaOiabfs5aenaaBaaaleaacqWGNbWzaeqaaOGaeiykaKIaeiilaWIaeeiiaaYaaabCaeaacqWGWbaCdaWgaaWcbaGaemyAaKgabeaakiabg2da9iabigdaXaWcbaGaemyAaKMaeyypa0JaeGymaedabaGaeGOmaidaniabggHiLdGccqGGSaalcaWLjaGaaCzcamaabmaabaGaeGOmaidacaGLOaGaayzkaaaaaa@5670@

where fi(Δg)=12πI0(κi)exp⁡(κicos⁡(Δg−μi))
 MathType@MTEF@5@5@+=feaafiart1ev1aaatCvAUfKttLearuWrP9MDH5MBPbIqV92AaeXatLxBI9gBaebbnrfifHhDYfgasaacH8akY=wiFfYdH8Gipec8Eeeu0xXdbba9frFj0=OqFfea0dXdd9vqai=hGuQ8kuc9pgc9s8qqaq=dirpe0xb9q8qiLsFr0=vr0=vr0dc8meaabaqaciaacaGaaeqabaqabeGadaaakeaacqWGMbGzdaWgaaWcbaGaemyAaKgabeaakiabcIcaOiabfs5aenaaBaaaleaacqWGNbWzaeqaaOGaeiykaKIaeyypa0ZaaSaaaeaacqaIXaqmaeaacqaIYaGmiiGacqWFapaCcqWGjbqsdaWgaaWcbaGaeGimaadabeaakiabcIcaOiab=P7aRnaaBaaaleaacqWGPbqAaeqaaOGaeiykaKcaaiGbcwgaLjabcIha4jabcchaWjabcIcaOiab=P7aRnaaBaaaleaacqWGPbqAaeqaaOGagi4yamMaei4Ba8Maei4CamNaeiikaGIaeuiLdq0aaSbaaSqaaiabdEgaNbqabaGccqGHsislcqWF8oqBdaWgaaWcbaGaemyAaKgabeaakiabcMcaPiabcMcaPaaa@563E@, *i *= 1, 2; 0 ≤ *κ*_*i*_; -*π *<*μ*_*i *_≤ *π*. Here, *p*_*i *_is the mixing parameter, *μ*_*i *_is the mean direction for distribution *i*, *κ*_*i *_is the concentration parameter characterizing the variability of the estimated differences Δ_*g *_about *μ*_*i*_, and *I*_0_(*κ*_*i*_)is the modified Bessel function of the first kind and order zero. We expect that one von Mises distribution has mean close to 0 radians, because it consists of a concordant subset of genes having the same phase in the two tissues, whereas the other distribution contains a set of "discordant" genes. The variation in shift characterizing genes of the second set can be measured by summing1 - cos(Δ_*g*_) [11].

The log likelihood for the mixture of two von Mises distributions in (2) is

L(Δ;θ)=∑g=1nlog⁡e(∑i=12pi2πI0(κi)exp⁡(κicos⁡(Δg−μi))).     (3)
 MathType@MTEF@5@5@+=feaafiart1ev1aaatCvAUfKttLearuWrP9MDH5MBPbIqV92AaeXatLxBI9gBaebbnrfifHhDYfgasaacH8akY=wiFfYdH8Gipec8Eeeu0xXdbba9frFj0=OqFfea0dXdd9vqai=hGuQ8kuc9pgc9s8qqaq=dirpe0xb9q8qiLsFr0=vr0=vr0dc8meaabaqaciaacaGaaeqabaqabeGadaaakeaacqWGmbatcqGGOaakcqqHuoarcqGG7aWoiiGacqWF4oqCcqGGPaqkcqGH9aqpdaaeWbqaaiGbcYgaSjabc+gaVjabcEgaNnaaBaaaleaacqWGLbqzaeqaaaqaaiabdEgaNjabg2da9iabigdaXaqaaiabd6gaUbqdcqGHris5aOWaaeWaaeaadaaeWbqaamaalaaabaGaemiCaa3aaSbaaSqaaiabdMgaPbqabaaakeaacqaIYaGmcqaHapaCcqWGjbqsdaWgaaWcbaGaeGimaadabeaakiabcIcaOiab=P7aRnaaBaaaleaacqWGPbqAaeqaaOGaeiykaKcaaiGbcwgaLjabcIha4jabcchaWnaabmaabaGae8NUdS2aaSbaaSqaaiabdMgaPbqabaGccyGGJbWycqGGVbWBcqGGZbWCcqGGOaakcqqHuoardaWgaaWcbaGaem4zaCgabeaakiabgkHiTiab=X7aTnaaBaaaleaacqWGPbqAaeqaaOGaeiykaKcacaGLOaGaayzkaaaaleaacqWGPbqAcqGH9aqpcqaIXaqmaeaacqaIYaGma0GaeyyeIuoaaOGaayjkaiaawMcaaiabc6caUiaaxMaacaWLjaWaaeWaaeaacqaIZaWmaiaawIcacaGLPaaaaaa@70CE@

The parameters in the vector (*p*_1_, *κ*_1_, *μ*_1_, *κ*_2_, *μ*_2_) in the mixture model (3) can be estimated using the Newton-type optimization method in the Matlab optimzation toolbox. To ensure convergence to the global solution, we use fifty random starting points. A comparison of the performance of various estimators can be found in [15]. We chose the Newton-type optimization method in the estimation due to its simplicity and flexibility of converting unconstrained searching to constrained optimization by adding constraints on the mixing parameter *p*_*1*_, i.e., 0.15 <*p*_*1 *_< 0.85 or the concentration parameter *κ*_1_, or *κ*_2_, i.e., *κ*_1 _<10 and *κ*_2 _< 10. Upon obtaining the five estimated parameters (p^1,κ^1,μ^1,κ^2,μ^2
 MathType@MTEF@5@5@+=feaafiart1ev1aaatCvAUfKttLearuWrP9MDH5MBPbIqV92AaeXatLxBI9gBaebbnrfifHhDYfgasaacH8akY=wiFfYdH8Gipec8Eeeu0xXdbba9frFj0=OqFfea0dXdd9vqai=hGuQ8kuc9pgc9s8qqaq=dirpe0xb9q8qiLsFr0=vr0=vr0dc8meaabaqaciaacaGaaeqabaqabeGadaaakeaacuWGWbaCgaqcamaaBaaaleaacqaIXaqmaeqaaOGaeiilaWccciGaf8NUdSMbaKaadaWgaaWcbaGaeGymaedabeaakiabcYcaSiqb=X7aTzaajaWaaSbaaSqaaiabigdaXaqabaGccqGGSaalcuWF6oWAgaqcamaaBaaaleaacqaIYaGmaeqaaOGaeiilaWIaf8hVd0MbaKaadaWgaaWcbaGaeGOmaidabeaaaaa@3E65@) in the mixture model (2), we statistically assign each of the Δ_*g *_to one of the two components based on its relative likelihood. That is, gene *g *is assigned to cluster 1 if p^1f^1(Δg)>p^2f^2(Δg)
 MathType@MTEF@5@5@+=feaafiart1ev1aaatCvAUfKttLearuWrP9MDH5MBPbIqV92AaeXatLxBI9gBaebbnrfifHhDYfgasaacH8akY=wiFfYdH8Gipec8Eeeu0xXdbba9frFj0=OqFfea0dXdd9vqai=hGuQ8kuc9pgc9s8qqaq=dirpe0xb9q8qiLsFr0=vr0=vr0dc8meaabaqaciaacaGaaeqabaqabeGadaaakeaacuWGWbaCgaqcamaaBaaaleaacqaIXaqmaeqaaOGafmOzayMbaKaadaWgaaWcbaGaeGymaedabeaakiabcIcaOiabfs5aenaaBaaaleaacqWGNbWzaeqaaOGaeiykaKIaeyOpa4JafmiCaaNbaKaadaWgaaWcbaGaeGOmaidabeaakiqbdAgaMzaajaWaaSbaaSqaaiabikdaYaqabaGccqGGOaakcqqHuoardaWgaaWcbaGaem4zaCgabeaakiabcMcaPaaa@4156@, otherwise to cluster 2.

### Circular-circular regression

In a recent article [9] we described the notion of association between the phase angles of a set of cell-cycle genes from a pair of experiments using the circular-circular regression model of Downs and Mardia [14]. Within each cluster obtained above, we shall apply the methodology described in [9] to examine the association between the estimated phase angles of the genes in the two tissues.

Consider a pair of angular random variables for cluster *i *as ( φ^igy
 MathType@MTEF@5@5@+=feaafiart1ev1aaatCvAUfKttLearuWrP9MDH5MBPbIqV92AaeXatLxBI9gBaebbnrfifHhDYfgasaacH8akY=wiFfYdH8Gipec8Eeeu0xXdbba9frFj0=OqFfea0dXdd9vqai=hGuQ8kuc9pgc9s8qqaq=dirpe0xb9q8qiLsFr0=vr0=vr0dc8meaabaqaciaacaGaaeqabaqabeGadaaakeaaiiGacuWFgpGzgaqcamaaDaaaleaacqWGPbqAcqWGNbWzaeaacqWG5bqEaaaaaa@32D6@, φ^igx
 MathType@MTEF@5@5@+=feaafiart1ev1aaatCvAUfKttLearuWrP9MDH5MBPbIqV92AaeXatLxBI9gBaebbnrfifHhDYfgasaacH8akY=wiFfYdH8Gipec8Eeeu0xXdbba9frFj0=OqFfea0dXdd9vqai=hGuQ8kuc9pgc9s8qqaq=dirpe0xb9q8qiLsFr0=vr0=vr0dc8meaabaqaciaacaGaaeqabaqabeGadaaakeaaiiGacuWFgpGzgaqcamaaDaaaleaacqWGPbqAcqWGNbWzaeaacqWG4baEaaaaaa@32D4@), *i *= 1, 2, *g *= 1, ..., *n*, with mean directions *α*_*i *_and *β*_*i*_, respectively. Further, suppose *η*_*ig *_denotes the mean direction of φ^igy
 MathType@MTEF@5@5@+=feaafiart1ev1aaatCvAUfKttLearuWrP9MDH5MBPbIqV92AaeXatLxBI9gBaebbnrfifHhDYfgasaacH8akY=wiFfYdH8Gipec8Eeeu0xXdbba9frFj0=OqFfea0dXdd9vqai=hGuQ8kuc9pgc9s8qqaq=dirpe0xb9q8qiLsFr0=vr0=vr0dc8meaabaqaciaacaGaaeqabaqabeGadaaakeaaiiGacuWFgpGzgaqcamaaDaaaleaacqWGPbqAcqWGNbWzaeaacqWG5bqEaaaaaa@32D6@ given φ^igx
 MathType@MTEF@5@5@+=feaafiart1ev1aaatCvAUfKttLearuWrP9MDH5MBPbIqV92AaeXatLxBI9gBaebbnrfifHhDYfgasaacH8akY=wiFfYdH8Gipec8Eeeu0xXdbba9frFj0=OqFfea0dXdd9vqai=hGuQ8kuc9pgc9s8qqaq=dirpe0xb9q8qiLsFr0=vr0=vr0dc8meaabaqaciaacaGaaeqabaqabeGadaaakeaaiiGacuWFgpGzgaqcamaaDaaaleaacqWGPbqAcqWGNbWzaeaacqWG4baEaaaaaa@32D4@. In the present context, this would be the mean estimated phase angle of a gene in one tissue, conditional on its estimated phase angle in the other tissue. Downs and Mardia [16] introduced the following flexible circular regression model to regress φ^igy
 MathType@MTEF@5@5@+=feaafiart1ev1aaatCvAUfKttLearuWrP9MDH5MBPbIqV92AaeXatLxBI9gBaebbnrfifHhDYfgasaacH8akY=wiFfYdH8Gipec8Eeeu0xXdbba9frFj0=OqFfea0dXdd9vqai=hGuQ8kuc9pgc9s8qqaq=dirpe0xb9q8qiLsFr0=vr0=vr0dc8meaabaqaciaacaGaaeqabaqabeGadaaakeaaiiGacuWFgpGzgaqcamaaDaaaleaacqWGPbqAcqWGNbWzaeaacqWG5bqEaaaaaa@32D6@ on φ^igx
 MathType@MTEF@5@5@+=feaafiart1ev1aaatCvAUfKttLearuWrP9MDH5MBPbIqV92AaeXatLxBI9gBaebbnrfifHhDYfgasaacH8akY=wiFfYdH8Gipec8Eeeu0xXdbba9frFj0=OqFfea0dXdd9vqai=hGuQ8kuc9pgc9s8qqaq=dirpe0xb9q8qiLsFr0=vr0=vr0dc8meaabaqaciaacaGaaeqabaqabeGadaaakeaaiiGacuWFgpGzgaqcamaaDaaaleaacqWGPbqAcqWGNbWzaeaacqWG4baEaaaaaa@32D4@

tan⁡ηig−βi2=ωitan⁡φ^igx−αi2,     (4)
 MathType@MTEF@5@5@+=feaafiart1ev1aaatCvAUfKttLearuWrP9MDH5MBPbIqV92AaeXatLxBI9gBaebbnrfifHhDYfgasaacH8akY=wiFfYdH8Gipec8Eeeu0xXdbba9frFj0=OqFfea0dXdd9vqai=hGuQ8kuc9pgc9s8qqaq=dirpe0xb9q8qiLsFr0=vr0=vr0dc8meaabaqaciaacaGaaeqabaqabeGadaaakeaacyGG0baDcqGGHbqycqGGUbGBdaWcaaqaaGGaciab=D7aOnaaBaaaleaacqWGPbqAcqWGNbWzaeqaaOGaeyOeI0Iae8NSdi2aaSbaaSqaaiabdMgaPbqabaaakeaacqaIYaGmaaGaeyypa0Jae8xYdC3aaSbaaSqaaiabdMgaPbqabaGccyGG0baDcqGGHbqycqGGUbGBdaWcaaqaaiqb=z8aMzaajaWaa0baaSqaaiabdMgaPjabdEgaNbqaaiabdIha4baakiabgkHiTiab=f7aHnaaBaaaleaacqWGPbqAaeqaaaGcbaGaeGOmaidaaiabcYcaSiaaxMaacaWLjaWaaeWaaeaacqaI0aanaiaawIcacaGLPaaaaaa@52E5@

where *ω*_*i *_denotes the "slope" parameter of the regression and *η*_*ig *_is the mean direction of φ^igy
 MathType@MTEF@5@5@+=feaafiart1ev1aaatCvAUfKttLearuWrP9MDH5MBPbIqV92AaeXatLxBI9gBaebbnrfifHhDYfgasaacH8akY=wiFfYdH8Gipec8Eeeu0xXdbba9frFj0=OqFfea0dXdd9vqai=hGuQ8kuc9pgc9s8qqaq=dirpe0xb9q8qiLsFr0=vr0=vr0dc8meaabaqaciaacaGaaeqabaqabeGadaaakeaaiiGacuWFgpGzgaqcamaaDaaaleaacqWGPbqAcqWGNbWzaeaacqWG5bqEaaaaaa@32D6@ conditional on φ^igx
 MathType@MTEF@5@5@+=feaafiart1ev1aaatCvAUfKttLearuWrP9MDH5MBPbIqV92AaeXatLxBI9gBaebbnrfifHhDYfgasaacH8akY=wiFfYdH8Gipec8Eeeu0xXdbba9frFj0=OqFfea0dXdd9vqai=hGuQ8kuc9pgc9s8qqaq=dirpe0xb9q8qiLsFr0=vr0=vr0dc8meaabaqaciaacaGaaeqabaqabeGadaaakeaaiiGacuWFgpGzgaqcamaaDaaaleaacqWGPbqAcqWGNbWzaeaacqWG4baEaaaaaa@32D4@. The above model allows for estimating not only the rotational angle *θ*_*i *_= *β*_*i *_-*α*_*i*_, but also the slope parameter *ω*_*i*_. As in Downs and Mardia [16], to avoid multiple solutions, we restrict - 1 ≤ *ω*_*i *_≤ 1 and -*π *≤ *α*_*i *_≤ *π *and -*π *≤ *β*_*i *_≤ *π*. We model the conditional distribution of φ^igy
 MathType@MTEF@5@5@+=feaafiart1ev1aaatCvAUfKttLearuWrP9MDH5MBPbIqV92AaeXatLxBI9gBaebbnrfifHhDYfgasaacH8akY=wiFfYdH8Gipec8Eeeu0xXdbba9frFj0=OqFfea0dXdd9vqai=hGuQ8kuc9pgc9s8qqaq=dirpe0xb9q8qiLsFr0=vr0=vr0dc8meaabaqaciaacaGaaeqabaqabeGadaaakeaaiiGacuWFgpGzgaqcamaaDaaaleaacqWGPbqAcqWGNbWzaeaacqWG5bqEaaaaaa@32D6@ given φ^igx
 MathType@MTEF@5@5@+=feaafiart1ev1aaatCvAUfKttLearuWrP9MDH5MBPbIqV92AaeXatLxBI9gBaebbnrfifHhDYfgasaacH8akY=wiFfYdH8Gipec8Eeeu0xXdbba9frFj0=OqFfea0dXdd9vqai=hGuQ8kuc9pgc9s8qqaq=dirpe0xb9q8qiLsFr0=vr0=vr0dc8meaabaqaciaacaGaaeqabaqabeGadaaakeaaiiGacuWFgpGzgaqcamaaDaaaleaacqWGPbqAcqWGNbWzaeaacqWG4baEaaaaaa@32D4@ as a von Mises with concentration parameter κic
 MathType@MTEF@5@5@+=feaafiart1ev1aaatCvAUfKttLearuWrP9MDH5MBPbIqV92AaeXatLxBI9gBaebbnrfifHhDYfgasaacH8akY=wiFfYdH8Gipec8Eeeu0xXdbba9frFj0=OqFfea0dXdd9vqai=hGuQ8kuc9pgc9s8qqaq=dirpe0xb9q8qiLsFr0=vr0=vr0dc8meaabaqaciaacaGaaeqabaqabeGadaaakeaaiiGacqWF6oWAdaqhaaWcbaGaemyAaKgabaGaem4yamgaaaaa@313C@, i.e.,

φigy|φigx∼M(ηig(φigx;αi,βi,ωi)κic).     (5)
 MathType@MTEF@5@5@+=feaafiart1ev1aaatCvAUfKttLearuWrP9MDH5MBPbIqV92AaeXatLxBI9gBaebbnrfifHhDYfgasaacH8akY=wiFfYdH8Gipec8Eeeu0xXdbba9frFj0=OqFfea0dXdd9vqai=hGuQ8kuc9pgc9s8qqaq=dirpe0xb9q8qiLsFr0=vr0=vr0dc8meaabaqaciaacaGaaeqabaqabeGadaaakeaaiiGacqWFgpGzdaqhaaWcbaGaemyAaKMaem4zaCgabaGaemyEaKhaaOGaeiiFaWNae8NXdy2aa0baaSqaaiabdMgaPjabdEgaNbqaaiabdIha4baakiablYJi6iabd2eannaabmaabaGaeq4TdG2aaSbaaSqaaiabdMgaPjabdEgaNbqabaGccqGGOaakcqWFgpGzdaqhaaWcbaGaemyAaKMaem4zaCgabaGaemiEaGhaaOGaei4oaSJae8xSde2aaSbaaSqaaiabdMgaPbqabaGccqGGSaalcqWFYoGydaWgaaWcbaGaemyAaKgabeaakiabcYcaSiab=L8a3naaBaaaleaacqWGPbqAaeqaaOGaeiykaKIae8NUdS2aa0baaSqaaiabdMgaPbqaaiabdogaJbaaaOGaayjkaiaawMcaaiabc6caUiaaxMaacaWLjaWaaeWaaeaacqaI1aqnaiaawIcacaGLPaaaaaa@603D@

As shown in Downs and Mardia [16], the angular error φ^igy
 MathType@MTEF@5@5@+=feaafiart1ev1aaatCvAUfKttLearuWrP9MDH5MBPbIqV92AaeXatLxBI9gBaebbnrfifHhDYfgasaacH8akY=wiFfYdH8Gipec8Eeeu0xXdbba9frFj0=OqFfea0dXdd9vqai=hGuQ8kuc9pgc9s8qqaq=dirpe0xb9q8qiLsFr0=vr0=vr0dc8meaabaqaciaacaGaaeqabaqabeGadaaakeaaiiGacuWFgpGzgaqcamaaDaaaleaacqWGPbqAcqWGNbWzaeaacqWG5bqEaaaaaa@32D6@ -*η*_*ig*_(φ^igx
 MathType@MTEF@5@5@+=feaafiart1ev1aaatCvAUfKttLearuWrP9MDH5MBPbIqV92AaeXatLxBI9gBaebbnrfifHhDYfgasaacH8akY=wiFfYdH8Gipec8Eeeu0xXdbba9frFj0=OqFfea0dXdd9vqai=hGuQ8kuc9pgc9s8qqaq=dirpe0xb9q8qiLsFr0=vr0=vr0dc8meaabaqaciaacaGaaeqabaqabeGadaaakeaaiiGacuWFgpGzgaqcamaaDaaaleaacqWGPbqAcqWGNbWzaeaacqWG4baEaaaaaa@32D4@;*α*_*i*_, *β*_*i*_, *ω*_*i*_) is von Mises with mean 0 and concentration parameter *κ*_*i*_, where

ηig(φ^igx;αi,βi,ωi)=βi+2tan⁡−1(ωitan⁡12(φ^igx−αi)).     (6)
 MathType@MTEF@5@5@+=feaafiart1ev1aaatCvAUfKttLearuWrP9MDH5MBPbIqV92AaeXatLxBI9gBaebbnrfifHhDYfgasaacH8akY=wiFfYdH8Gipec8Eeeu0xXdbba9frFj0=OqFfea0dXdd9vqai=hGuQ8kuc9pgc9s8qqaq=dirpe0xb9q8qiLsFr0=vr0=vr0dc8meaabaqaciaacaGaaeqabaqabeGadaaakeaaiiGacqWF3oaAdaWgaaWcbaGaemyAaKMaem4zaCgabeaakmaabmaabaGaf8NXdyMbaKaadaqhaaWcbaGaemyAaKMaem4zaCgabaGaemiEaGhaaOGaei4oaSJae8xSde2aaSbaaSqaaiabdMgaPbqabaGccqGGSaalcqWFYoGydaWgaaWcbaGaemyAaKgabeaakiabcYcaSiab=L8a3naaBaaaleaacqWGPbqAaeqaaaGccaGLOaGaayzkaaGaeyypa0Jae8NSdi2aaSbaaSqaaiabdMgaPbqabaGccqGHRaWkcqaIYaGmcyGG0baDcqGGHbqycqGGUbGBdaahaaWcbeqaaiabgkHiTiabigdaXaaakmaabmaabaGae8xYdC3aaSbaaSqaaiabdMgaPbqabaGccyGG0baDcqGGHbqycqGGUbGBdaWcaaqaaiabigdaXaqaaiabikdaYaaadaqadaqaaiqb=z8aMzaajaWaa0baaSqaaiabdMgaPjabdEgaNbqaaiabdIha4baakiabgkHiTiab=f7aHnaaBaaaleaacqWGPbqAaeqaaaGccaGLOaGaayzkaaaacaGLOaGaayzkaaGaeiOla4IaaCzcaiaaxMaadaqadaqaaiabiAda2aGaayjkaiaawMcaaaaa@6D06@

The association from one tissue to the other of genes in each cluster (φ^iy
 MathType@MTEF@5@5@+=feaafiart1ev1aaatCvAUfKttLearuWrP9MDH5MBPbIqV92AaeXatLxBI9gBaebbnrfifHhDYfgasaacH8akY=wiFfYdH8Gipec8Eeeu0xXdbba9frFj0=OqFfea0dXdd9vqai=hGuQ8kuc9pgc9s8qqaq=dirpe0xb9q8qiLsFr0=vr0=vr0dc8meaabaqaciaacaGaaeqabaqabeGadaaakeaaiiGacuWFgpGzgaqcamaaDaaaleaacqWGPbqAaeaacqWG5bqEaaaaaa@317F@, φ^ix
 MathType@MTEF@5@5@+=feaafiart1ev1aaatCvAUfKttLearuWrP9MDH5MBPbIqV92AaeXatLxBI9gBaebbnrfifHhDYfgasaacH8akY=wiFfYdH8Gipec8Eeeu0xXdbba9frFj0=OqFfea0dXdd9vqai=hGuQ8kuc9pgc9s8qqaq=dirpe0xb9q8qiLsFr0=vr0=vr0dc8meaabaqaciaacaGaaeqabaqabeGadaaakeaaiiGacuWFgpGzgaqcamaaDaaaleaacqWGPbqAaeaacqWG4baEaaaaaa@317D@), *i *= 1, 2, can then be assessed using the *F*-test derived by Downs and Mardia [16].

### A bootstrap test for number of clusters

To assess whether there are two clusters in the mixture of Δ_*g*_, *g *= 1,..., *n*, in (1), in the following we propose a bootstrap methodology to test the null hypothesis that Δ_*g *_'s are a random sample from a single von-Mises distribution against the alternative hypothesis that they are from a mixture of two independent von-Mises distributions.

Let cv=∑g=1n(1−cos⁡(rg))
 MathType@MTEF@5@5@+=feaafiart1ev1aaatCvAUfKttLearuWrP9MDH5MBPbIqV92AaeXatLxBI9gBaebbnrfifHhDYfgasaacH8akY=wiFfYdH8Gipec8Eeeu0xXdbba9frFj0=OqFfea0dXdd9vqai=hGuQ8kuc9pgc9s8qqaq=dirpe0xb9q8qiLsFr0=vr0=vr0dc8meaabaqaciaacaGaaeqabaqabeGadaaakeaacqWGJbWycqWG2bGDcqGH9aqpdaaeWbqaaiabcIcaOiabigdaXiabgkHiTiGbcogaJjabc+gaVjabcohaZjabcIcaOiabdkhaYnaaBaaaleaacqWGNbWzaeqaaOGaeiykaKIaeiykaKcaleaacqWGNbWzcqGH9aqpcqaIXaqmaeaacqWGUbGBa0GaeyyeIuoaaaa@43C9@ denote an estimate of the circular variance for the combined sample of *n *= *n*_1 _+ *n*_2 _observations, based on residuals from a single circular regression, while cv1=∑g=1n1(1−cos⁡(rg))
 MathType@MTEF@5@5@+=feaafiart1ev1aaatCvAUfKttLearuWrP9MDH5MBPbIqV92AaeXatLxBI9gBaebbnrfifHhDYfgasaacH8akY=wiFfYdH8Gipec8Eeeu0xXdbba9frFj0=OqFfea0dXdd9vqai=hGuQ8kuc9pgc9s8qqaq=dirpe0xb9q8qiLsFr0=vr0=vr0dc8meaabaqaciaacaGaaeqabaqabeGadaaakeaacqWGJbWycqWG2bGDdaWgaaWcbaGaeGymaedabeaakiabg2da9maaqahabaGaeiikaGIaeGymaeJaeyOeI0Iagi4yamMaei4Ba8Maei4CamNaeiikaGIaemOCai3aaSbaaSqaaiabdEgaNbqabaGccqGGPaqkcqGGPaqkaSqaaiabdEgaNjabg2da9iabigdaXaqaaiabd6gaUnaaBaaameaacqaIXaqmaeqaaaqdcqGHris5aaaa@460C@ and cv2=∑g=1n2(1−cos⁡(rg))
 MathType@MTEF@5@5@+=feaafiart1ev1aaatCvAUfKttLearuWrP9MDH5MBPbIqV92AaeXatLxBI9gBaebbnrfifHhDYfgasaacH8akY=wiFfYdH8Gipec8Eeeu0xXdbba9frFj0=OqFfea0dXdd9vqai=hGuQ8kuc9pgc9s8qqaq=dirpe0xb9q8qiLsFr0=vr0=vr0dc8meaabaqaciaacaGaaeqabaqabeGadaaakeaacqWGJbWycqWG2bGDdaWgaaWcbaGaeGOmaidabeaakiabg2da9maaqahabaGaeiikaGIaeGymaeJaeyOeI0Iagi4yamMaei4Ba8Maei4CamNaeiikaGIaemOCai3aaSbaaSqaaiabdEgaNbqabaGccqGGPaqkcqGGPaqkaSqaaiabdEgaNjabg2da9iabigdaXaqaaiabd6gaUnaaBaaameaacqaIYaGmaeqaaaqdcqGHris5aaaa@4610@ denote the estimates of the circular variances for the two individual clusters separately based on residuals from two circular regressions.

The proposed bootstrap procedure is described in the following steps:

1) Regress phase angles in *y *tissue on phase angles in *x *tissue using the circular-circular regression model (4) and compute the circular variance *cv *based on the residuals *r*_*g*_, *g *= 1, ..., *n*, from this single circular regression;

2) Compute for each gene g the difference Δ_*g *_= φ^gy
 MathType@MTEF@5@5@+=feaafiart1ev1aaatCvAUfKttLearuWrP9MDH5MBPbIqV92AaeXatLxBI9gBaebbnrfifHhDYfgasaacH8akY=wiFfYdH8Gipec8Eeeu0xXdbba9frFj0=OqFfea0dXdd9vqai=hGuQ8kuc9pgc9s8qqaq=dirpe0xb9q8qiLsFr0=vr0=vr0dc8meaabaqaciaacaGaaeqabaqabeGadaaakeaaiiGacuWFgpGzgaqcamaaDaaaleaacqWGNbWzaeaacqWG5bqEaaaaaa@317B@ - φ^gx
 MathType@MTEF@5@5@+=feaafiart1ev1aaatCvAUfKttLearuWrP9MDH5MBPbIqV92AaeXatLxBI9gBaebbnrfifHhDYfgasaacH8akY=wiFfYdH8Gipec8Eeeu0xXdbba9frFj0=OqFfea0dXdd9vqai=hGuQ8kuc9pgc9s8qqaq=dirpe0xb9q8qiLsFr0=vr0=vr0dc8meaabaqaciaacaGaaeqabaqabeGadaaakeaaiiGacuWFgpGzgaqcamaaDaaaleaacqWGNbWzaeaacqWG4baEaaaaaa@3179@, where φ^gy
 MathType@MTEF@5@5@+=feaafiart1ev1aaatCvAUfKttLearuWrP9MDH5MBPbIqV92AaeXatLxBI9gBaebbnrfifHhDYfgasaacH8akY=wiFfYdH8Gipec8Eeeu0xXdbba9frFj0=OqFfea0dXdd9vqai=hGuQ8kuc9pgc9s8qqaq=dirpe0xb9q8qiLsFr0=vr0=vr0dc8meaabaqaciaacaGaaeqabaqabeGadaaakeaaiiGacuWFgpGzgaqcamaaDaaaleaacqWGNbWzaeaacqWG5bqEaaaaaa@317B@ and φ^gx
 MathType@MTEF@5@5@+=feaafiart1ev1aaatCvAUfKttLearuWrP9MDH5MBPbIqV92AaeXatLxBI9gBaebbnrfifHhDYfgasaacH8akY=wiFfYdH8Gipec8Eeeu0xXdbba9frFj0=OqFfea0dXdd9vqai=hGuQ8kuc9pgc9s8qqaq=dirpe0xb9q8qiLsFr0=vr0=vr0dc8meaabaqaciaacaGaaeqabaqabeGadaaakeaaiiGacuWFgpGzgaqcamaaDaaaleaacqWGNbWzaeaacqWG4baEaaaaaa@3179@ are the estimated phase angles for gene *g *in tissues *y *and *x*;

3) Fit the phase differences Δ_*g*_, *g *= 1, ..., *n*, to a two-component mixture of von-Mises model, obtaining two separate clusters with *n*_1 _and *n*_2 _genes in provisional clusters 1 and 2, respectively;

4) Regress *n*_1 _and *n*_2 _phase angles of *y*_1 _on *x*_1 _and *y*_2 _on *x*_2 _separately for the two clusters, and obtain the residuals *r*^*cluster*1 ^and *r*^*cluster*2 ^from each of the two regressions;

5) Compute the circular variances *cv*_1 _and *cv*_2 _for each of the two-cluster sets of residuals *r*^*cluster*1 ^and *r*^*cluster*2 ^from the regressions carried out in step 4);

6) Calculate the test statistic: *T *= *cv *- *cv*_1 _- *cv*_2_

7) Compute the absolute values of *r*_*g *_obtained from Step 1, and randomly assign a +/- sign to each *r*_*g*_, call it rg*
 MathType@MTEF@5@5@+=feaafiart1ev1aaatCvAUfKttLearuWrP9MDH5MBPbIqV92AaeXatLxBI9gBaebbnrfifHhDYfgasaacH8akY=wiFfYdH8Gipec8Eeeu0xXdbba9frFj0=OqFfea0dXdd9vqai=hGuQ8kuc9pgc9s8qqaq=dirpe0xb9q8qiLsFr0=vr0=vr0dc8meaabaqaciaacaGaaeqabaqabeGadaaakeaacqWGYbGCdaqhaaWcbaGaem4zaCgabaGaeiOkaOcaaaaa@3079@, then obtain bootstrapped data rgbootstrap
 MathType@MTEF@5@5@+=feaafiart1ev1aaatCvAUfKttLearuWrP9MDH5MBPbIqV92AaeXatLxBI9gBaebbnrfifHhDYfgasaacH8akY=wiFfYdH8Gipec8Eeeu0xXdbba9frFj0=OqFfea0dXdd9vqai=hGuQ8kuc9pgc9s8qqaq=dirpe0xb9q8qiLsFr0=vr0=vr0dc8meaabaqaciaacaGaaeqabaqabeGadaaakeaacqWGYbGCdaqhaaWcbaGaem4zaCgabaGaemOyaiMaem4Ba8Maem4Ba8MaemiDaqNaem4CamNaemiDaqNaemOCaiNaemyyaeMaemiCaahaaaaa@3C2A@ from rg*
 MathType@MTEF@5@5@+=feaafiart1ev1aaatCvAUfKttLearuWrP9MDH5MBPbIqV92AaeXatLxBI9gBaebbnrfifHhDYfgasaacH8akY=wiFfYdH8Gipec8Eeeu0xXdbba9frFj0=OqFfea0dXdd9vqai=hGuQ8kuc9pgc9s8qqaq=dirpe0xb9q8qiLsFr0=vr0=vr0dc8meaabaqaciaacaGaaeqabaqabeGadaaakeaacqWGYbGCdaqhaaWcbaGaem4zaCgabaGaeiOkaOcaaaaa@3079@;

8) Obtain each pseudo phase angle for the heart data by *η*_*g *_+ rgbootstrap
 MathType@MTEF@5@5@+=feaafiart1ev1aaatCvAUfKttLearuWrP9MDH5MBPbIqV92AaeXatLxBI9gBaebbnrfifHhDYfgasaacH8akY=wiFfYdH8Gipec8Eeeu0xXdbba9frFj0=OqFfea0dXdd9vqai=hGuQ8kuc9pgc9s8qqaq=dirpe0xb9q8qiLsFr0=vr0=vr0dc8meaabaqaciaacaGaaeqabaqabeGadaaakeaacqWGYbGCdaqhaaWcbaGaem4zaCgabaGaemOyaiMaem4Ba8Maem4Ba8MaemiDaqNaem4CamNaemiDaqNaemOCaiNaemyyaeMaemiCaahaaaaa@3C2A@ => φgy,bootstrap
 MathType@MTEF@5@5@+=feaafiart1ev1aaatCvAUfKttLearuWrP9MDH5MBPbIqV92AaeXatLxBI9gBaebbnrfifHhDYfgasaacH8akY=wiFfYdH8Gipec8Eeeu0xXdbba9frFj0=OqFfea0dXdd9vqai=hGuQ8kuc9pgc9s8qqaq=dirpe0xb9q8qiLsFr0=vr0=vr0dc8meaabaqaciaacaGaaeqabaqabeGadaaakeaaiiGacqWFgpGzdaqhaaWcbaGaem4zaCgabaGaemyEaKNaeiilaWIaemOyaiMaem4Ba8Maem4Ba8MaemiDaqNaem4CamNaemiDaqNaemOCaiNaemyyaeMaemiCaahaaaaa@3ED8@, where *η*_*g *_is the predicted angle in tissue *y *based on regression performed in Step 1;

9) Repeat the loop from step 1) to 8) 3000 times using φ^gx
 MathType@MTEF@5@5@+=feaafiart1ev1aaatCvAUfKttLearuWrP9MDH5MBPbIqV92AaeXatLxBI9gBaebbnrfifHhDYfgasaacH8akY=wiFfYdH8Gipec8Eeeu0xXdbba9frFj0=OqFfea0dXdd9vqai=hGuQ8kuc9pgc9s8qqaq=dirpe0xb9q8qiLsFr0=vr0=vr0dc8meaabaqaciaacaGaaeqabaqabeGadaaakeaaiiGacuWFgpGzgaqcamaaDaaaleaacqWGNbWzaeaacqWG4baEaaaaaa@3179@, the bootstrap data θgy,bootstrap
 MathType@MTEF@5@5@+=feaafiart1ev1aaatCvAUfKttLearuWrP9MDH5MBPbIqV92AaeXatLxBI9gBaebbnrfifHhDYfgasaacH8akY=wiFfYdH8Gipec8Eeeu0xXdbba9frFj0=OqFfea0dXdd9vqai=hGuQ8kuc9pgc9s8qqaq=dirpe0xb9q8qiLsFr0=vr0=vr0dc8meaabaqaciaacaGaaeqabaqabeGadaaakeaaiiGacqWF4oqCdaqhaaWcbaGaem4zaCgabaGaemyEaKNaeiilaWIaemOyaiMaem4Ba8Maem4Ba8MaemiDaqNaem4CamNaemiDaqNaemOCaiNaemyyaeMaemiCaahaaaaa@3ED5@ to replace φ^gy
 MathType@MTEF@5@5@+=feaafiart1ev1aaatCvAUfKttLearuWrP9MDH5MBPbIqV92AaeXatLxBI9gBaebbnrfifHhDYfgasaacH8akY=wiFfYdH8Gipec8Eeeu0xXdbba9frFj0=OqFfea0dXdd9vqai=hGuQ8kuc9pgc9s8qqaq=dirpe0xb9q8qiLsFr0=vr0=vr0dc8meaabaqaciaacaGaaeqabaqabeGadaaakeaaiiGacuWFgpGzgaqcamaaDaaaleaacqWGNbWzaeaacqWG5bqEaaaaaa@317B@ and replacing *r*_*g *_by rgbootstrap
 MathType@MTEF@5@5@+=feaafiart1ev1aaatCvAUfKttLearuWrP9MDH5MBPbIqV92AaeXatLxBI9gBaebbnrfifHhDYfgasaacH8akY=wiFfYdH8Gipec8Eeeu0xXdbba9frFj0=OqFfea0dXdd9vqai=hGuQ8kuc9pgc9s8qqaq=dirpe0xb9q8qiLsFr0=vr0=vr0dc8meaabaqaciaacaGaaeqabaqabeGadaaakeaacqWGYbGCdaqhaaWcbaGaem4zaCgabaGaemOyaiMaem4Ba8Maem4Ba8MaemiDaqNaem4CamNaemiDaqNaemOCaiNaemyyaeMaemiCaahaaaaa@3C2A@. For the bootstrap data denote the T in step 6 by *T*^*bootstrap*^.

Then the bootstrap *p*-value is the proportion of *T*^*bootstrap *^that are greater than the calculated value of test statistic *T*.

## Results

### Datasets

We apply our methodologies to 52 circadian-related cycling transcripts that are expressed in mouse heart and liver tissues, identified by Storch *et al*. [2]. In Storch's studies, mice were entrained to a 12 hrs light/dark cycle for more than two weeks, then placed in a constant dim light for more than 42 hrs. The tissue samples were taken from sacrificed mice at 4-hour intervals for 48 hrs, or about two circadian cycles, as in the circadian studies of Panda *et al*. [1].

**Figure 1:**
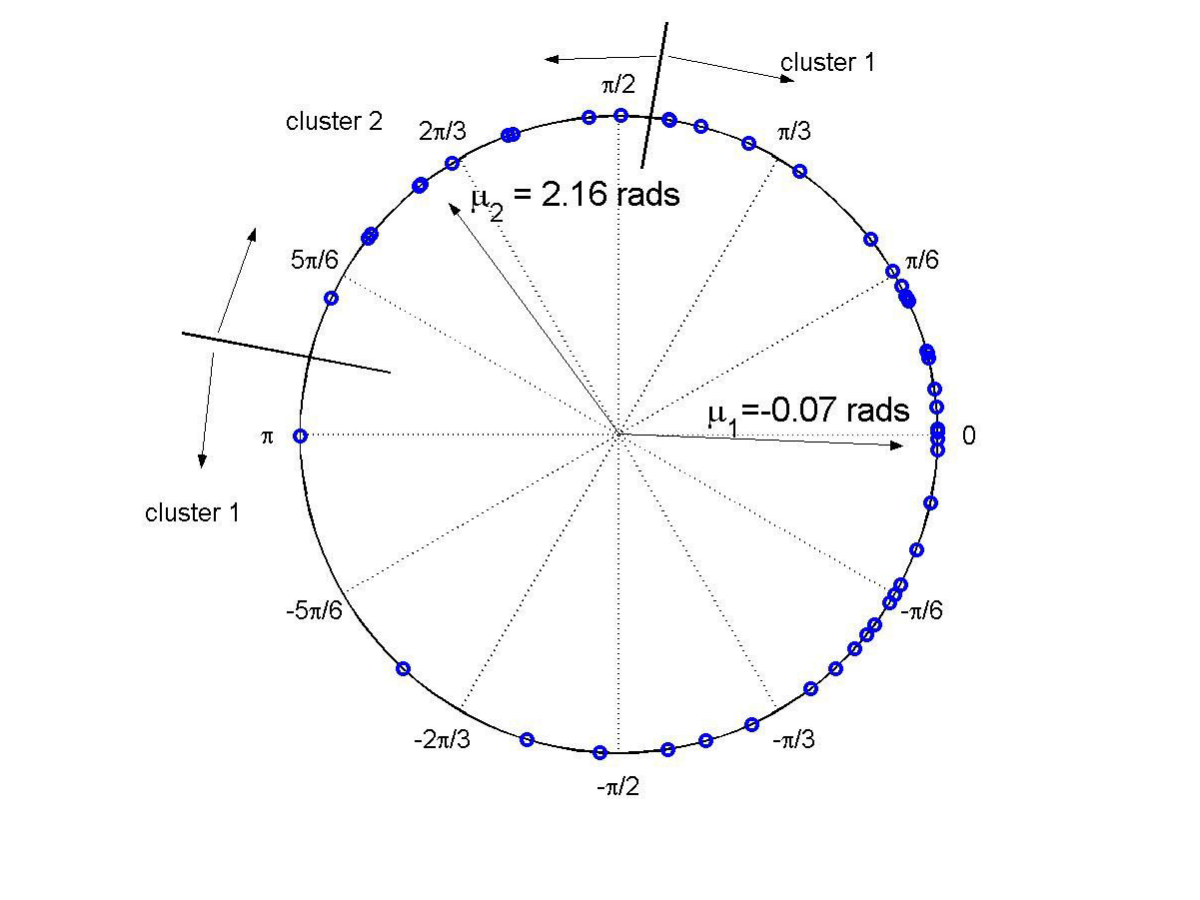
Plot of Δ_*g *_showing clusters for 48 circadian-related cycling transcripts [2].

Due to the poor fit of our random-periods model [13] to the expression of four transcripts (accession numbers: AI834950, AF003348, AF043288, and AB014494), we excluded them from the list of 52 circadian-related transcripts [2] in our analysis. The estimated phase angles for the remaining 48 transcripts in both heart and liver for clusters 1 and 2 are listed in Tables 1 and 2, respectively. The angular differences (heart, denoted as *y*, minus liver, denoted as *x*) Δ_*g *_are plotted in Figure 1.

**Table 1:** Estimates of phase angles of circadian-related transcripts in heart and liver [2] of cluster 1

Probe ID	Gene Description	Accession	Phase in heart (rads)	Phase in liver (rads)
98079_at	carbonic anhydrase 14	AB005450	0.12	0.61
93184_at	expressed sequence C76179	AI596362	0.27	0.95
92821_at	ubiquitin specific protease 2	AF079565	0.29	-2.85
94549_at	RIKEN cDNA 1200003O06 gene	AI315650	0.30	0.67
160795_at	expressed sequence AW122395	AW123662	0.31	-0.13
101539_f_at	carboxylesterase 3	AW226939	0.34	0.08
93619_at	period homolog (Drosophila)	AF022992	0.35	2.67
97473_at	transmembrane 4 superfamily member 7	AW124470	0.58	1.72
100581_at	cystatin B	U59807	0.62	1.45
97556_at	cerebellar ataxia 3	AI843178	1.60	1.59
94145_at	interferon beta, fibroblast	K00020	2.35	-2.51
160195_at	RIKEN cDNA 1200013P24 gene	AI846961	2.62	-2.92
96102_i_at	RAD23b homolog (S. cerevisiae)	X92411	2.83	1.42
97485_at	RIKEN cDNA 1200015P13 gene	AI850953	-3.06	2.74
96774_at	expressed sequence AA960555	AW047139	-2.86	2.88
101002_at	ornithine decarboxylase antizyme inhibitor	AF032128	-2.77	-3.01
104241_at	expressed sequence AA408983	AI854379	-2.69	-2.69
104200_at		AW048729	-2.57	3.05
102797_at	retinal short-chain dehydrogenase/reductase 1X95281		-2.56	-2.35
98478_at	cyclin G2	U95826	-2.45	2.68
100323_at	S-adenosylmethionine decarboxylase 2	Z23077	-2.43	-2.86
97808_at	splicing factor 3b, subunit 1, 155 kDa	AI844532	-2.37	-2.51
92264_at	SRY-box containing gene 3	X94125	-2.18	2.69
93874_s_at	interleukin 11 receptor, alpha chain 2	U69491	-2.16	-2.11
96852_at	protein kinase, cAMP dependent regulatory, type I alpha	AW122197	-2.04	-1.48
103957_at	transferrin receptor	X57349	-1.61	-2.06
96793_at	RIKEN cDNA 1500016M21 gene	AI607813	-1.32	-2.63
94461_at	pre-B-cell colony-enhancing factor	AI852144	-1.22	-1.49
93694_at	period homolog 2 (Drosophila)	AF036893	-0.84	-0.83
93121_at	ribosomal protein S24	X60289	-0.77	0.86
98892_at	lipin 1	AI846934	-0.38	0.26
98766_at	SH3-domain binding protein 5 (BTK-associated	AB016835	-0.36	1.50
95614_at	chromobox homolog 5 (Drosophila HP1a)	AI852086	-0.26	1.03
104320_at	pyridoxal (pyridoxine, vitamin B6) kinase	AI841777	-0.19	0.33
94944_at	protein kinase, cAMP dependent regulatory, type I beta	AW125016	-0.18	-1.15
99076_at	thyroid hormone receptor alpha	U09504	-0.13	-0.21
95057_at	homocysteine-inducible, endoplasmic reticulum stress-inducible ubiquintin-like domain member 1	AI846938	-0.12	-0.55
160489_at	tumor necrosis factor, alpha-induced protein	L24118	-0.02	0.91

1 radian = 57.3 degrees.

**Table 2:** Estimates of phase angles of circadian-related transcripts in both heart and liver [2] of cluster 2

Probe ID	Descriptions of Genes	Accession	Phase in heart (rads)	Phase in liver (rads)
101007_at	MAP kinase-interacting serine/threonine kinase 2	AI845732	0.08	-2.16
100555_at	Down syndrome critical region homolog 1 (human)	AI846152	3.01	0.55
93324_at	zinc finger protein 36, C3H type-like 1	M58566	3.05	0.93
92820_at	ubiquitin specific protease 2	AI846522	-3.06	1.31
97224_at	proline-rich nuclear receptorcoactivator I	AI851394	-2.72	1.31
100126_at	chromatin accessibility complex I	AA967263	-1.54	-3.10
102955_at	nuclear factor, interleukin 3, regulated	U83148	-1.21	2.59
95307_at	similar to scavenger receptor cysteine rich domain containing group B	AW047736	-0.62	-2.55
160117_at	thyrotroph embryonic factor	AI850638	-0.28	-2.97
160841_at	D site albumin promoter binding protein	AW047343	-0.09	-1.76

**Figure 2:**
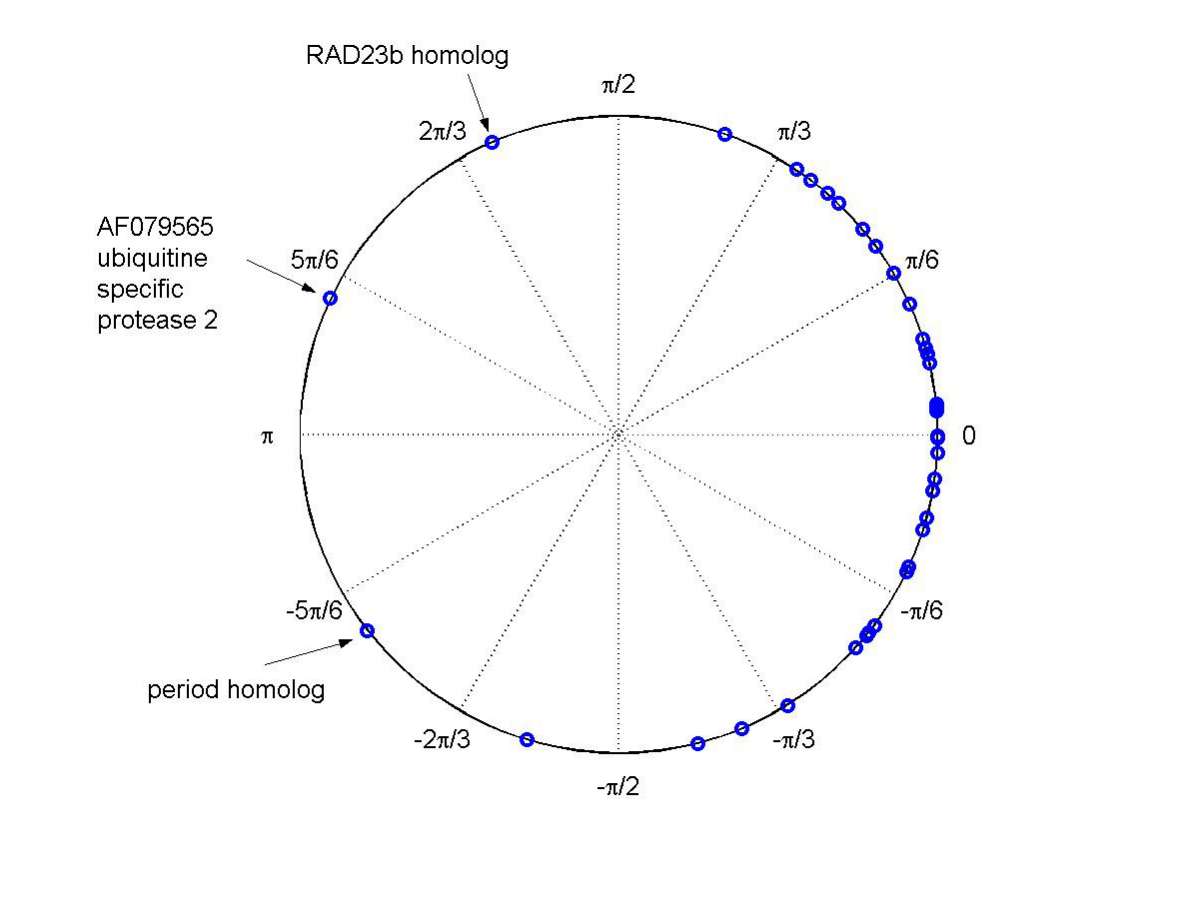
Residual plot of circular-circular regression for the 38 synchronized genes in heart and liver tissues [2] from cluster 1.

**Figure 3:**
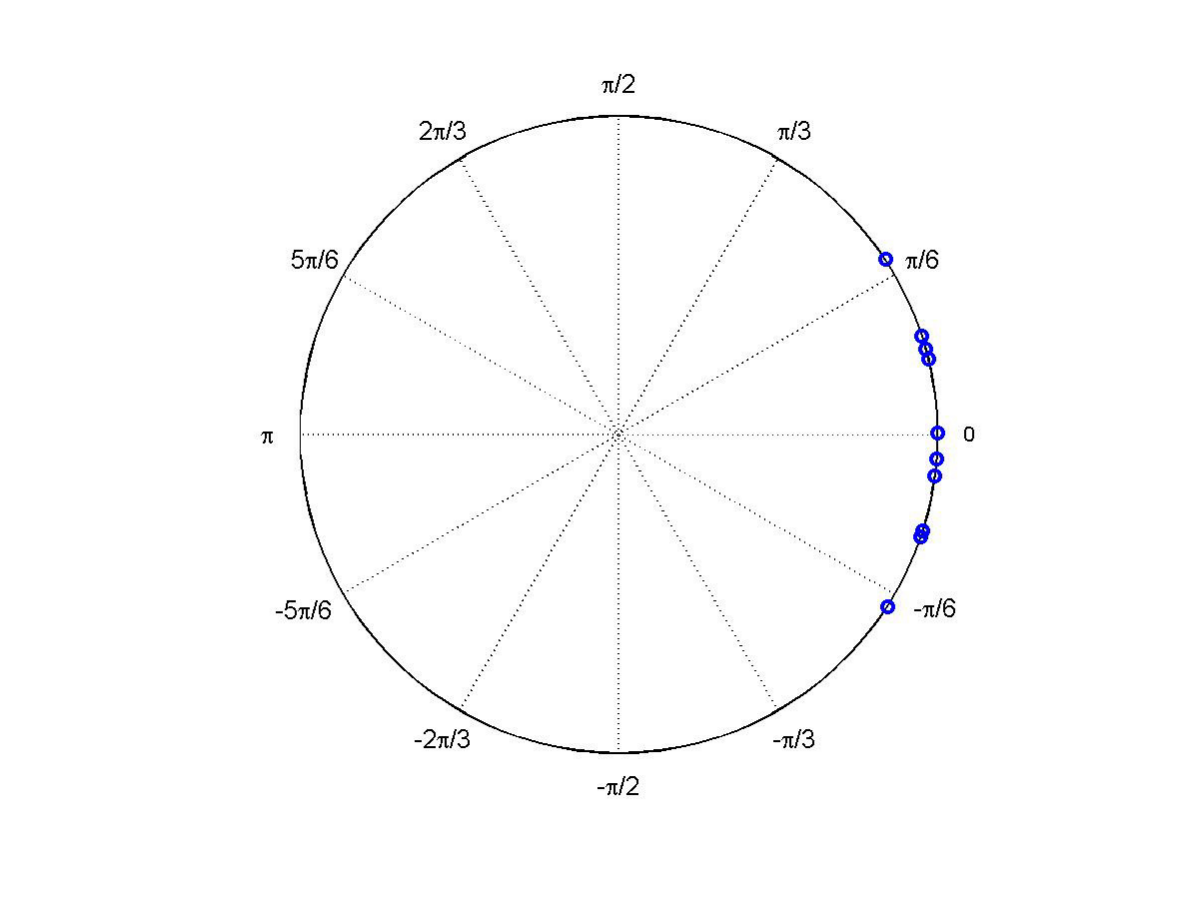
Residual plot of circular-circular regression for the 10 discordant genes in heart and liver tissues [2] from cluster 2.

The 48 circadian-related cycling transcripts were assigned into two clusters based on a mixture of two von Mises distributions, as described above. The first cluster contains 38 genes (Table 1) and the second cluster contains 10 genes (Table 2). The five estimated parameters (p^1,κ^1,μ^1,κ^2,μ^2
 MathType@MTEF@5@5@+=feaafiart1ev1aaatCvAUfKttLearuWrP9MDH5MBPbIqV92AaeXatLxBI9gBaebbnrfifHhDYfgasaacH8akY=wiFfYdH8Gipec8Eeeu0xXdbba9frFj0=OqFfea0dXdd9vqai=hGuQ8kuc9pgc9s8qqaq=dirpe0xb9q8qiLsFr0=vr0=vr0dc8meaabaqaciaacaGaaeqabaqabeGadaaakeaacuWGWbaCgaqcamaaBaaaleaacqaIXaqmaeqaaOGaeiilaWccciGaf8NUdSMbaKaadaWgaaWcbaGaeGymaedabeaakiabcYcaSiqb=X7aTzaajaWaaSbaaSqaaiabigdaXaqabaGccqGGSaalcuWF6oWAgaqcamaaBaaaleaacqaIYaGmaeqaaOGaeiilaWIaf8hVd0MbaKaadaWgaaWcbaGaeGOmaidabeaaaaa@3E65@) = (0.79, 1.50, -0.07, 4.56, 2.16). The distance between the two clusters in mean direction (μ^1−μ^2
 MathType@MTEF@5@5@+=feaafiart1ev1aaatCvAUfKttLearuWrP9MDH5MBPbIqV92AaeXatLxBI9gBaebbnrfifHhDYfgasaacH8akY=wiFfYdH8Gipec8Eeeu0xXdbba9frFj0=OqFfea0dXdd9vqai=hGuQ8kuc9pgc9s8qqaq=dirpe0xb9q8qiLsFr0=vr0=vr0dc8meaabaqaciaacaGaaeqabaqabeGadaaakeaaiiGacuWF8oqBgaqcamaaBaaaleaacqaIXaqmaeqaaOGaeyOeI0Iaf8hVd0MbaKaadaWgaaWcbaGaeGOmaidabeaaaaa@336B@) is 2.23 rads, suggesting that the ten transcripts in cluster 2 have different points of peak expression in heart and liver. This can also be seen in Figures 2 and 3. The mean direction of 38 phase angles in the cluster 1 is -0.07 rads, suggesting that the peak expression times for the 38 cycling genes in heart and liver are close to synchronized. In contrast, the peak expression times for the 10 genes in heart and liver (in cluster 2) are away from the 0 direction by 2.16 rads, with heart ahead of liver by about 8 hrs. This result suggests that these 10 discordant circadian-related genes may play different roles in the heart and liver.

**Figure 4:**
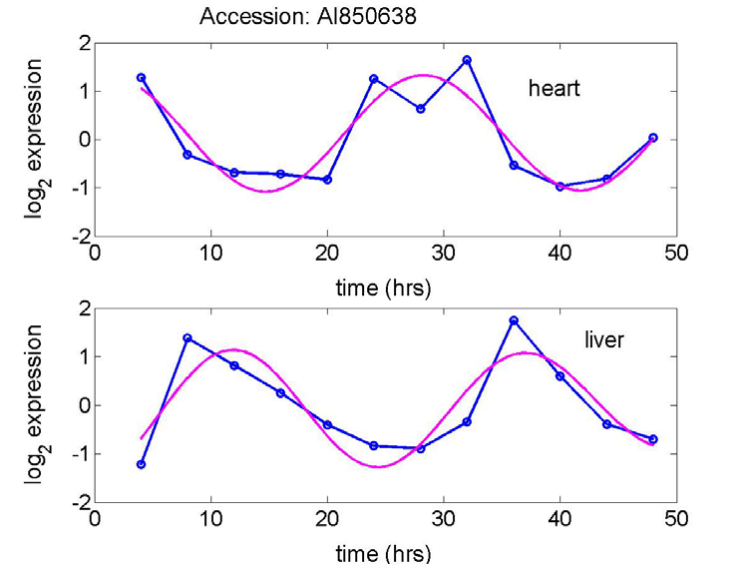
Plots of log_2 _expression for the transcript AI850638 in heart and liver tissues [2]. Data (-o-) and fitted curve of the model (—). The phase difference in the heart and liver is -0.28 rads - (-2.97) rads = 2.69 rads.

**Figure 5:**
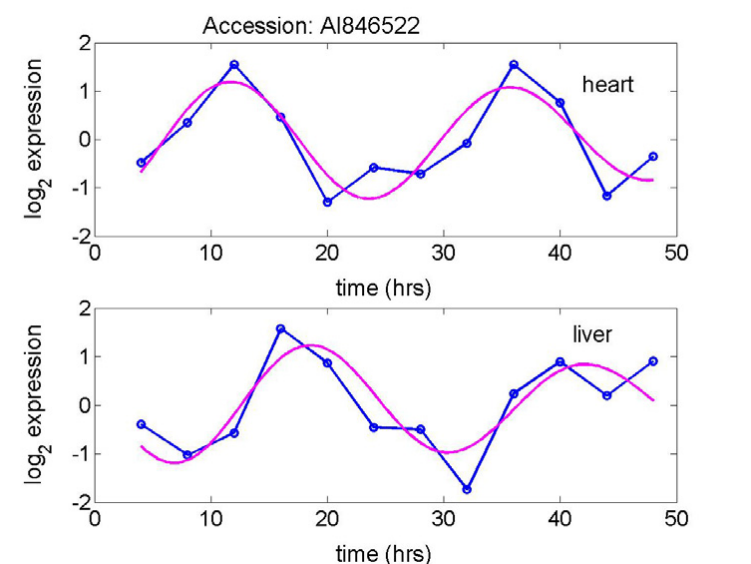
Plots of log_2 _expression for the transcript AI846522 in heart and liver tissues [2]. Data (-o-) and fitted curve of the model (—). The phase difference of the gene in the heart and liver is -3.06 rads - 1.31 rads = 1.9 rads (module).

We estimated the across-tissue association of the genes in each of the two clusters by regressing the phase angles in heart, denoted as *y*, onto those in liver, denoted as *x*, using the circular-circular regression model described above (5). The estimated rotational parameters, the slope, and the concentration parameter for the von Mises distribution are (α^1,β^1,ω^1,κ^1c
 MathType@MTEF@5@5@+=feaafiart1ev1aaatCvAUfKttLearuWrP9MDH5MBPbIqV92AaeXatLxBI9gBaebbnrfifHhDYfgasaacH8akY=wiFfYdH8Gipec8Eeeu0xXdbba9frFj0=OqFfea0dXdd9vqai=hGuQ8kuc9pgc9s8qqaq=dirpe0xb9q8qiLsFr0=vr0=vr0dc8meaabaqaciaacaGaaeqabaqabeGadaaakeaaiiGacuWFXoqygaqcamaaBaaaleaacqaIXaqmaeqaaOGaeiilaWIaf8NSdiMbaKaadaWgaaWcbaGaeGymaedabeaakiabcYcaSiqb=L8a3zaajaWaaSbaaSqaaiabigdaXaqabaGccqGGSaalcuWF6oWAgaqcamaaDaaaleaacqaIXaqmaeaacqWGJbWyaaaaaa@3C21@) = (-0.83, -0.91, 0.58, 1.85) for cluster 1 and (α^2,β^2,ω^2,κ^2c
 MathType@MTEF@5@5@+=feaafiart1ev1aaatCvAUfKttLearuWrP9MDH5MBPbIqV92AaeXatLxBI9gBaebbnrfifHhDYfgasaacH8akY=wiFfYdH8Gipec8Eeeu0xXdbba9frFj0=OqFfea0dXdd9vqai=hGuQ8kuc9pgc9s8qqaq=dirpe0xb9q8qiLsFr0=vr0=vr0dc8meaabaqaciaacaGaaeqabaqabeGadaaakeaaiiGacuWFXoqygaqcamaaBaaaleaacqaIYaGmaeqaaOGaeiilaWIaf8NSdiMbaKaadaWgaaWcbaGaeGOmaidabeaakiabcYcaSiqb=L8a3zaajaWaaSbaaSqaaiabikdaYaqabaGccqGGSaalcuWF6oWAgaqcamaaDaaaleaacqaIYaGmaeaacqWGJbWyaaaaaa@3C29@) = (-2.85, -0.76, 0.86, 9.41) for cluster 2. Figure 2 shows the residuals for all genes, i.e. the angular difference between the phase angle in heart and the prediction based on the same gene's pattern of expression in liver. The residuals for the 10 genes in the second cluster are shown in Figure 3. We can see that the activation times of these transcripts in heart and liver tissues are matched well after a 2.16 radian clockwise rotation of the phase angles in heart relative to in liver. Upon obtaining the 'slope parameters' ω^1
 MathType@MTEF@5@5@+=feaafiart1ev1aaatCvAUfKttLearuWrP9MDH5MBPbIqV92AaeXatLxBI9gBaebbnrfifHhDYfgasaacH8akY=wiFfYdH8Gipec8Eeeu0xXdbba9frFj0=OqFfea0dXdd9vqai=hGuQ8kuc9pgc9s8qqaq=dirpe0xb9q8qiLsFr0=vr0=vr0dc8meaabaqaciaacaGaaeqabaqabeGadaaakeaaiiGacuWFjpWDgaqcamaaBaaaleaacqaIXaqmaeqaaaaa@2FAC@ and ω^2
 MathType@MTEF@5@5@+=feaafiart1ev1aaatCvAUfKttLearuWrP9MDH5MBPbIqV92AaeXatLxBI9gBaebbnrfifHhDYfgasaacH8akY=wiFfYdH8Gipec8Eeeu0xXdbba9frFj0=OqFfea0dXdd9vqai=hGuQ8kuc9pgc9s8qqaq=dirpe0xb9q8qiLsFr0=vr0=vr0dc8meaabaqaciaacaGaaeqabaqabeGadaaakeaaiiGacuWFjpWDgaqcamaaBaaaleaacqaIYaGmaeqaaaaa@2FAE@, we tested the null hypothesis H_0_: *ω*_*i *_= 0 vs. the alternative hypothesis, H_a_: *ω*_*i *_≠ 0, *i *= 1, 2, using the test statistic derived by Downs and Mardia [14]. The corresponding *p*-values for clusters 1 and 2 were less than 5 × 10^-7 ^and 1.4 × 10^-4^, respectively, suggesting that the associations of the circadian activation times in heart and liver are strong in both clusters. Here we included the expression plots of the transcripts AI850638 and AI846522 in heart and liver tissues [2] and the fits of our model to the two transcripts in Figures 4 and 5, accordingly. The plots reveal that the fits of our model to the data are reasonably good, and peak times of the two transcripts in liver tissue are markedly lagged relative to heart tissue.

Based on 3000 bootstraps using the procedure outlined in section of bootstrap test, we found that the estimated *p*-value for sufficiency of a one-component distribution in the phase difference of heart and liver tissues for the 48 genes was 0.063. Although this is not significant at 5% level of significance, it suggests that a two-component von-Mises distribution for the phase in heart and liver tissues better describes the relationships among the peak expression times for the studied genes.

## Discussion and conclusion

Our analysis of the peak expression times (phases) for a set of 48-circadian-related genes expressed in both heart and liver tissues [2] suggests that not all of the genes are maximally expressed at the same time in the two tissues. Instead, among the 48 genes, 38 are synchronized in phase or peak expression times in heart and liver tissues, and the other 10 genes express earlier by about 2.23 rads or 8 hours in heart than in liver. Our bootstrapping test result supports, albeit weakly, the existence of two distinct subsets among the 48 genes. Although our findings are based on the single experimental dataset of Storch *et al*. [2], our results are similar to an earlier observation made by Panda *et al*. [1] that the peak expression times for some genes are not synchronized in suprachiasmatic nuclei (SCN) compared to liver. One implication of our results, giving quantitative support for the conclusion of Storch *et al*. [2], may be that some commonly expressed circadian-related genes may perform different functions across different organs [1,2].

We have developed a new bootstrap method for assessing the adequacy of one versus the need for two clusters of genes in the two sets of phase angles in heart and liver tissues for the 48 genes. In particular, we evaluated the significance of the circular variances of the residuals from circular-circular regression of the phase angles in heart on in liver in one cluster vs. two clusters. In contrast, most studies on mixtures of circular variables have focused on dividing a set of data into two subsets. To the best of our knowledge, no studies have used a mixture of two components for circular datasets for testing heterogeneity of a cyclic pattern.

This work would not have been undertaken without the interesting observations by Panda *et al*. [1], Storch *et al*. [2] and Ueda *et al*. [3] that a few circadian-related genes expressed out of phase across two tissues of mouse. In addition, quantitatively the results of our statistical analysis depend on the approximation of sinusoidal waveform for circadian gene expression, on reasonably accurate estimation of the phase angles, on a relative large sample size of genes common to two tissues, on the approximate validity of the von Mises distribution for each cluster of differences. In previous circadian gene expression studies [1-3], a tissue sample was commonly taken at each 4-hr interval for two circadian cycles, i.e., 12 time points per gene expression. Although our fits to the 48 circadian gene expression in both tissues are reasonably good, as shown in Figures 4, 5, further experimental and simulation studies may be needed to understand the role of sample size and sampling frequency on phase estimation when a sinusoidal waveform is presumed for circadian rhythm. In this work, we considered the difference of two phases for a gene in two tissues as a random variable modeled by a von Mises distribution on a circle. The corresponding uncertainties are captured by the degree to which the von Mises distribution is spread out on the circle.

The 48 circadian related genes expressed in heart and liver of mouse each provide a pair of peak expression times. We have assumed that there are at most two clusters. Further experimental studies are needed for testing whether there might be more than two clusters with for 48 genes. While our method can hypothetically be extended to allow one to test the need for 3 clusters rather than for 2 clusters, the sample size of 48 genes, may not be sufficient to carry out such a test with much power. The results of the two-clusters analysis must be regarded as descriptive. Our analysis of circadian gene expression may serve to stimulate further methodological development in circular/directional statistical analysis of genes that may be expressed differently in phase in two or more tissues. The genes in each cluster would need to be scrutinized separately for further elucidation of their tissue-specific biological and physiological functions [1,2,5].

Our methodologies can be, in principle, extended to analyzing multiple circadian gene expression data sets across multiple tissues (organs), e.g., kidney, heart, liver, and SCN where investigators are interested in understanding whether there is one set of core circadian-related genes that are similar in their patterns of activation across different organs (or tissues), and others that are differently expressed in different tissues, as suggested by Reppert and Weaver [5]. Because the maximum likelihood approach considered in this paper may become challenging due to computational complexity, further methodology development is needed in this area. For such multiple mixture problems, one may want to consider a Bayes or empirical Bayes approach. However, to the best of our knowledge, Bayes and empirical Bayes methods for mixture problems associated with circular data are not well developed. The present application provides an excellent opportunity for developing such methodology for mixture problems associated with circular data. Secondly, using the standard likelihood approach, we clustered the 48 genes into two clusters on the basis of the phase difference between the two tissues. It would be interesting and useful to derive an estimate of "reliability" of clustering for each gene. One possible approach is to perform a bootstrap by selecting a simple random sample of 48 genes from the list of 48 genes and classify them into two clusters using procedure described in this paper. This procedure could be repeated for a large number of times, say 1000. One then could estimate the proportion of times a gene was classified into one of two clusters. Unfortunately, such a procedure does not work well when the number of genes is small and their "true" cluster memberships are unknown and the numbers of genes in each cluster are highly unbalanced. Nonetheless, these important questions need to be addressed because the number of potential applications for such a procedure is ever growing.

## Authors' contributions

DL conceived of the study, performed the calculations, and drafted the manuscript. SDP and CRW suggested the bootstrap method. SDP, CRW, and LL drafted the manuscript. All authors read and approved the final manuscript.
